# Should governments moralize health?

**DOI:** 10.1111/nyas.70030

**Published:** 2025-09-03

**Authors:** Steven R. Kraaijeveld

**Affiliations:** ^1^ Department of Ethics, Law, & Medical Humanities Amsterdam University Medical Centers Amsterdam The Netherlands

**Keywords:** ethics of moralization, government moralization, health moralization, health policy, moralization, public health ethics

## Abstract

Health is often moralized not only by individuals, but also by governments, which was particularly conspicuous during the COVID‐19 pandemic. This paper addresses the ethics of whether governments should moralize health. It first introduces a definition of moralizing health. It then distinguishes between different ways of moralizing health that affect its moral acceptability, including negative or positive framing, as well as different potential targets toward which moralizing may be directed: (1) persons, (2) behavior, or (3) society. It concludes that targeting individual persons and behavior, especially negatively, is morally unacceptable. Positive moralizing about a healthy society by governments may be more acceptable, but important conditions remain. Governments should not single out individuals or groups and moralizing should not be excessive. Moralized health outcomes should be equally achievable by all citizens and implementation of moral frames should be grounded in robust evidence that it will bring about desired outcomes (i.e., a healthier society) without being harmful or counterproductive. In short, governments should be very cautious about moralizing health. If one of the basic tasks of governments is to protect collective health, this is all too easily undermined by undue moralizing.

## Graphical abstract

This paper provides a discussion of the ethics of governments moralizing health that accounts for different ways of framing—negative or positive—as well as different potential targets: persons, behavior, or society. It argues that moralizing health becomes more acceptable as it moves away from singling out individuals and toward promoting positive collective health goals, even if important risks and conditions for its moral acceptability remain.

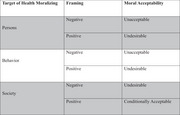

## INTRODUCTION

The COVID‐19 pandemic demonstrated just how quickly and profoundly health can be moralized. At the height of the outbreak, MIT Professor Emeritus Noam Chomsky stated that, when it comes to people who refuse to accept vaccines, “the right response for them is […] that they be isolated.” When asked how these ostracized unvaccinated citizens would obtain food during a pandemic, he added that “that's actually their problem.”^1^ That a highly regarded academic would publicly entertain the idea of allowing a group of citizens to potentially starve as a result of their medical choices reveals the extent to which other people's health behaviors can become subject to our moral judgments and moral condemnation.

Moralizing is ubiquitous in human life.^2^ Beyond anecdotal examples of public figures moralizing health, an emerging body of evidence suggests that COVID‐19 measures to prevent the spread of the virus, including vaccination, were widely associated with moral judgments, moral censure, and even discrimination of others.^3^, ^4^, ^5^, ^6^, ^7^ For example, one study on the impact of vaccination status on evaluations of individuals suspected of being sources of infection found that vaccinated participants—compared to unvaccinated participants—attributed greater responsibility to unvaccinated suspected sources and perceived them as being less moral, trustworthy, and empathetic.^8^ Another study analyzed cross‐sectional online surveys from eight countries (Denmark, Sweden, Germany, France, Italy, Hungary, the United Kingdom, and the United States) to examine whether complying with the advice of health authorities was moralized during the pandemic.^9^ The findings reveal that “large majorities find it justified to condemn those who do not keep a distance to others in public,” and that “around half of respondents blame ordinary citizens for the severity of the pandemic” (p. 257).^9^

The phenomenon of moralizing health extends far beyond the COVID‐19 pandemic. Health behavior is prone to becoming associated with moralizing judgments due to real and perceived risks that tend to be marked by uncertainty.^10^ Health risks can contribute to a setting of boundaries between “others who are dangerous,” for instance because they are infected with a disease or because they engage in health behavior deemed to be risky, “and the virtuous self” who avoids risks or who heeds dominant (i.e., commonly accepted) definitions of risk (p. 130).^11^ A classic example of a behavior that was once considered to be a matter of personal preference and discretion, but that has become increasingly moralized, is the smoking of cigarettes.^12^ Another example is when tuberculosis was finally understood to be an infectious rather than a hereditary disease, it entered the social–moral domain and became part of a wider process of “moralizing the microbe.”^13^ Obesity is another example of a health matter that is often linked to moral judgments.^14^, ^15^, ^16^ More indirect health‐related behavior and choices can also become moralized over time, like wearing a bicycle helmet or wearing one's seat belt.^17^ When health choices are moralized, they become more than mere choices; they come to function as loci for our normative judgments about the moral goodness and badness of behavior and other people.^12^, ^13^

There are high stakes involved when it comes to whether or not an issue is moralized. Moralization can fuel polarization, social strife, stigmatization, discrimination, and the dehumanization of others.^18^ Moralized obesity attitudes, for example, have been found to predict the endorsement of weight‐based discrimination.^19^ In general, moralized issues are more likely to (1) prompt scientific research, (2) receive funding from governments, (3) incite censure, (4) lead to motivated reasoning, (5) be transmitted from parents to children in the form of attitudes, and (6) become internalized as values.^20^ When a health issue becomes intertwined with moral judgments, this has far‐reaching consequences for individuals, groups, governments, and societies at large.^21^

At the same time, moralizing is not an unalloyed vice. Moralizing about social wrongs can lead to bad actors being held accountable for their actions^22^ and is generally associated with both negative and positive consequences.^23^ Moralizing (or even re‐moralizing) certain issues has been proposed as a remedy for perceived social, moral, and political ills.^24^ There is also a prima facie case to be made that at least some actions that affect the health of others, like deliberately infecting a person with a disease, are rightful targets of our moral judgments, including our moral condemnation. Still, moralizing measures and regulations can have pervasive negative social consequences,^25^, ^26^ which make it all the more important to analyze the phenomenon from an ethical perspective.^21^

Importantly, moralizing health is not limited to the actions of individuals. During the COVID‐19 pandemic, many governments and public health officials explicitly employed moral framing and moral messaging about infectious disease measures, thus effectively moralizing health. Examples include emphasizing people's moral responsibility with regard to personal relationships, specifically toward one's grandmother, while using both negative framing (“don't kill granny”)^27^ and positive framing (“protect ya grandma!”).^28^ This moral framing of infectious disease interventions furthermore ranged from targeting specific behavioral measures, like wearing face masks,^29^ to more general kinds of “we're all in this together” messaging drawing on moral notions of solidarity and collective responsibility.^30^

To date, the question of whether governments^a^ ought to moralize health has received scant philosophical attention. In particular, the ethics of moralizing health from the perspective of governments is highly understudied. Should governments employ moral frames when communicating with the public about health issues? How ought we to ethically evaluate governments moralizing health? These questions are crucial because framing is an increasingly important policy tool,^31^ and governments frequently employ appeals to morality and social norms—either overtly or tacitly—when it comes to health promotion and public health communications across a wide range of issues.^32^, ^33^, ^34^ Given that moralization can intensify polarization^35^ and has serious social and political consequences, it is all the more important to carefully consider the ethics of moralizing health.

This paper is structured as follows. In the first section, I offer a definition of moralizing health. One aim of the paper is to centralize the notion of moralizing health as a subject for ethical discussions. To that end, it is important to have a clear definition. While moralizing is often characterized as a vice within the relevant philosophical literature, I argue that moralizing health can also be understood more neutrally and, at least *prima facie*, as good in certain cases. This is important, because if moralizing were always a vice then that would arguably settle the ethics of moralizing health. I also propose two important distinctions for moralizing health: one between positive and negative ways of framing, and the other between different potential targets—(1) persons, (2) behavior, or (3) society more generally. In the second section of the paper, the preceding distinctions and arguments are applied directly to the question of whether governments ought to moralize health.

It should be noted that this paper will not be concerned either with the legality of other‐directed health behavior—with the question, for instance, of whether reckless infection of others ought to be criminalized^36^, ^37^—or more generally with the connection between morality and law.^38^ As important as legal questions are, I focus on the role of governments from an ethical rather than a legal perspective. The most contentious and polarized public debates about health issues tend, in any case, to center on moral concerns and judgments; for instance, about moral responsibility and blame for particular health choices and behaviors.^21^ Whether and how governments ought to participate in this discourse by actively moralizing health is the central question at stake.

## WHAT DOES IT MEAN TO MORALIZE HEALTH?

Before proceeding to a definition of moralizing health, something must be said about the potential moral value of health. For, if health either had no moral value or did not entail any morally relevant aspects, then it would be difficult to see why one should have any reason to moralize it.

### The moral value of health

Health has both inherent and instrumental value. Having good health—including the subjective experience of good health—is valuable not only in and of itself, but also for well‐being, obtaining valued goods, exercising autonomy, and pursuing various life goals.^39^ People value health in many different ways. There are myriad conceptions of health, which cannot possibly be covered here. For present purposes, I will adopt a practice‐oriented view of health according to which health is a thick concept that both describes a particular condition (i.e., a state of health) and evaluates that condition at the same time (e.g., as good or bad, desirable or undesirable).^40^ The evaluative component to health can explain not only people's subjective valuations of different states of health or illness, but also the normative judgments that are often associated with health or a lack of it—for example, that it is bad to be ill in the sense that it makes a person relatively worse off.

Normative judgments about health also extend beyond subjective valuations to judgments about the (potential) actions of other people. Our health is dependent on other people in many ways, meaning that others can make our health better or worse. Our health is also dependent more generally on the societies in which we live (e.g., on the quality and availability of health care). Given the dependencies of our health on other people and societal conditions, there are numerous ways in which our health can be harmed—and, in turn, ways in which we can harm the health of others. The possibility of harms to others through detrimental effects on others' health introduces a moral component, because not harming others (i.e., nonmaleficence) is a widely recognized moral imperative in moral philosophy and applied ethics, as well as various religions.^41^, ^42^

The notion of harm therefore often takes center stage in academic debates as well as interpersonal and societal conflicts about health. Health choices are, in general, more likely to be moralized when they are associated with harm to others.^43^ Vaccination provides a concrete example of an individual health choice that can potentially affect the health of others (e.g., by making one less likely to transmit disease), thus giving rise to a number of ethical considerations^44^, ^45^, ^46^ and potential moral reasons to act.^47^, ^48^,^b^

While others have argued that health itself has moral value,^49^ for instance because health is a basic human need,^50^ I do not commit to that position here. It is sufficient for my arguments in this paper to grant that individual health choices and behaviors are often associated with moral reasons to act (or not to act), both in theory and practice, if only through the moral imperative not to unduly harm others. This does not, of course, automatically justify moralizing health.^c^ Yet, it does reveal that there can be—at least in principle—a legitimate basis for moral judgments about some kinds of health behavior and health‐related choices.

Whether moralizing health by governments is ethically justified is, of course, another question, which will be addressed soon. Before doing so, it is important to clarify what exactly it means to moralize health.

### A definition of moralizing health

Moralizing often has strongly negative connotations both in common parlance and within the philosophical literature. According to one influential conceptualization, for instance, moralism “is a criticism leveled at people who are overly judgmental, self‐righteous, or unforgiving” (p. 342).^52^ Moralism and its cognates, like moralistic and moralizing are “primarily used pejoratively, both in and out of philosophy” (p. 251).^53^

Moralizing health can, of course, be vicious. For example, when politicians or government officials moralize an issue—including health—this can be done in disingenuous and highly questionable ways. Moralizing might be done in ways and for reasons that have been identified in related discussions as undesirable, self‐serving, or unethical—for instance, as objectionable forms of moral grandstanding,^54^ virtue signaling,^55^ political or group loyalty and out‐group exclusionary tactics,^56^, ^57^ illicit use of moral considerations,^58^ and so on. There are certainly many avenues through which moralizing can be a vice and steer people away from or even undermine morality.^52^

At the same time, it is all too easy to imagine a grave and ongoing social wrong—there are plenty of historical cases, and I leave it up to the reader to picture contemporary ones—that one might, at least on the surface, rightfully moralize, especially if the wrong in question has been insufficiently recognized (e.g., as rightfully falling within the scope of morality). It is not self‐evidently true that moralizing in a case like this would necessarily be wrong.

I therefore do not wish to adopt a wholly negative definition of moralizing. Instead, I want to stay close to a classic dictionary definition of the term. Merriam‐Webster^59^ provides two definitions of the transitive verb, *moralize*: (1) “to explain or interpret morally,” and (2) “to give a moral quality or direction to.” The second definition lends itself most immediately to moralizing health, and can be adapted in the following way: *To* *moralize health* *is* *to give a moral quality or direction to health*.

This formulation avoids defining the term in a way that, ironically, would moralize it. It preserves the possibility that moralizing is good or bad, useful or harmful, virtuous or vicious, and so on, given that the definition itself lacks a normative evaluation. As such, the question of whether it is good or bad to give a moral quality/direction to health—or, under which conditions it might be good or bad—remains open.

One important consequence of the above definition is that any framing of health in moral terms should be understood as moralizing health. Accordingly, discussions about health‐related moral framing, moral messaging, social norms communication, and so on, all fall within the purview of the concept of moralizing. This follows from the definition that to moralize health means giving it a moral quality/direction (which moral framing does).

Of course, whether the substantive moral content of a moralized claim is morally justified still needs to be determined.^21^ One condition that must hold for any instance of moralizing *X* is that *X* does, in fact, have a moral quality/direction. To this end, I follow Kraaijeveld and Jamrozik's approach^21^ to determining whether moralization is morally appropriate or not. For the purpose of this paper, and in the ensuing discussion, I assume that the moralizing of health in question is apt in the specific sense that the health matter that would be subject to moralizing does, in fact, have a moral quality (which, to be clear, does not in and of itself justify moralizing).

The question that naturally arises, after having established a case of moralizing health (e.g., in a particular public health message or moral frame), is whether or not this is morally acceptable or desirable. Before addressing that question directly, however, two morally relevant distinctions need to be made.

#### Distinction 1: Framing

The first distinction centers on the valence of the moralizing in question. While the definition provided above does not refer to whether the moral quality/direction is positive or negative, an issue can importantly be moralized in negative and positive ways. For any given health outcome *H*, it might be claimed that (1) it is good to bring about *H*, or (2) that it is bad to bring about *H*. The distinction here is between moral goodness (positive) and moral badness (negative), which are different ways of evaluating and assigning moral weight to an outcome.

The associated emotions that such framing stands to elicit are also likely to differ in important ways. When *H* is negatively framed, this will likely provoke negative reactive emotions in others (e.g., blame, disappointment, anger, disgust, resentment, and so on). When *H* is positively framed, this will likely produce positive reactive emotions in others (e.g., praise, approval, admiration, gratitude, and so on).

At a basic level, the core of a positively framed moralized health message might communicate: “engaging in (or abstaining from) health‐related behavior *B* is morally good).” Similarly, a negatively framed moralized health message might communicate: “engaging in (or abstaining from) health‐related behavior *B* is morally bad.”

Furthermore, the moral message may range from being wholly transparent and explicit (i.e., openly linking health with moral goodness or badness) to being opaque and implicit (i.e., merely suggesting a correlation between health and moral goodness or badness).^d^

Different ways of framing stand to bring about different responses and may cast a health issue—including relevant individuals or groups—in a good or bad (moral) light. As such, the distinction is ethically relevant and adds necessary complexity to discussions about the ethics of moralizing health.

#### Distinction 2: Target

The second distinction involves the target of moralizing. Historically, different loci for ethical analysis can be distinguished in ethics.^60^ Two common targets—that is, subjects or ends toward which ethical analysis may be aimed—include the *person* (or character) and the *behavior* (or action). Virtue ethics, for example, is traditionally concerned with human virtue or moral character,^61^ while consequentialist theories like utilitarianism concentrate on the desirability of outcomes associated with potential actions.^62^,^e^

Aside from persons and behavior, another target may be distinguished, namely, *society* (or the public/collective). Society is a target for ethical analysis within moral theories that involve moral perspectives about how societies ought to look or function—for example, for theories about social justice, moral notions of the common good, and so forth.^64^, ^65^ Just like a person or a particular behavior may be judged as morally good or bad, so a society or a collective outcome may be deemed morally good or bad.

Moral judgments about society do not necessarily need to refer to individual persons—even if a society is ultimately composed of individuals. For example, the hypothetical argument that a cooperative society is a morally good or a morally better society (e.g., compared to a noncooperative society) does not in itself specify any specific actors responsible for cooperation, nor any particular behaviors that would instantiate cooperation. However, even though it is theoretically possible to place moral value on society or on societal outcomes (like a good state of public health) without necessarily singling out individual actors or behaviors, in practice it will likely follow that people and behaviors are identified. If a cooperative society is considered to be a morally better society than a noncooperative one (as in the above example about cooperation), then noncooperative people and noncooperative behavior are, in all probability, going to be perceived as not contributing to the morally valued goal.

This relatively brief account of the different targets for ethical analysis is not intended to be exhaustive. It is chiefly meant to illustrate that, like ethical analysis itself, moralization may be directed toward different potential targets. In the case of moralizing health, this may primarily involve moral judgments about persons, behavior, or society more generally.

## SHOULD GOVERNMENTS MORALIZE HEALTH?

The two preceding distinctions not only allow us to more precisely address the question of whether governments ought to moralize health, but they also suggest that moralizing can be more or less ethically acceptable or desirable depending on different combinations of how it is framed and what it targets (see Table 1 for an overview). As such, I will treat each potential target in turn and consider whether, for that target, moralizing health either negatively or positively is ethically acceptable or desirable.

**TABLE 1:** An overview of the moral acceptability of governments moralizing health according to the target in question and different ways of framing.

Target of health moralizing	Framing	Moral acceptability
Persons	Negative	Unacceptable
	Positive	Undesirable
Behavior	Negative	Unacceptable
	Positive	Undesirable
Society	Negative	Undesirable
	Positive	Conditionally acceptable

### Persons

The first potential target of health moralizing is persons (or character). A general formulation of health moralizing that targets persons is as follows: Person(s) *P*(s) engaging in (or abstaining from) health‐related behavior *B* is/are morally good or bad.

There are numerous instances of government officials and politicians engaging in this kind of health moralizing during the COVID‐19 pandemic. Canadian Prime Minister Justin Trudeau's televised suggestion that the unvaccinated “are very often misogynistic and racist”^66^ is one prominent example of moralizing health by targeting persons through negative framing.^66^ It seems clear that this is an example of an unacceptable way of moralizing health. First of all, it inappropriately homogenizes a diverse group of people. Scholars have resisted associating a single term (like antivaxxer) with “the unambiguous identification of a specific clearly delineated referent or category of people,” pointing out that vaccination decisions “are made for many different reasons, may vary for particular types of vaccination, and over time,” so that individuals or groups “who are hesitant or refuse particular vaccinations do not conform to a common personality profile, attitudes, or homogenous set of beliefs.”^67^ Second of all, it singles out and impugns the (collective) character of this supposedly uniform group. It publicly shames citizens and makes them look (morally) bad on the basis of a claim for which there appears to be no evidence (none, in any case, was provided).

The purpose of Trudeau's suggestion seems to have been to rebuke persons who do not get vaccinated by aligning them with some of society's most unacceptable characters (i.e., misogynists and racists). Yet, even if such a claim was to motivate people to follow health measures (i.e., to get vaccinated), it is morally unacceptable that a government official would publicly target and stigmatize a group of citizens in this manner. When it comes to government policy, it is likewise morally unacceptable that health communications from official or representative sources would negatively frame the character of individual citizens or groups of citizens based on their health choices. As far as motivating behavioral change goes, there is substantial evidence that stigmatizing public health communication does not lead to good health outcomes; in fact, it often increases psychological reactance and has the opposite effect,^68^, ^69^, ^70^ which obviates any pragmatic arguments about negatively targeting persons.

What about positively targeting persons? It might seem that simply pointing out the characters of people who follow recommended health measures or live up to certain health ideals is morally unproblematic. After all, individuals are sometimes officially praised by governments for commendable acts, for example, through a medal of bravery, official recognition of community service, special contributions to a nation's standing, and so on. The praise of character does seem less immediately problematic than the denigration of character. However, for at least two reasons, health behaviors do not easily lend themselves to the kind of praise from governments that might be appropriate in other cases. First, it is rare for citizens to perform an especially morally praiseworthy health‐related act to the extent that it would warrant official government commendation. Second, governments and health organizations formulate rules for behavior through health policies, recommendations, guidelines, and so on, which people are generally expected to follow. If a health intervention is recommended for all, then why should any person or group of people receive praise for abiding by it?

If there is little room for singular praise, then perhaps governments might still opt to moralize adherence to health guidelines or health choices, like getting vaccinated, washing one's hands during flu season, or eating healthy food. Yet, this raises at least two issues. First, the argument quickly becomes absurd. Is the government supposed to praise citizens for every health‐related choice that it deems to be (morally) good? The consequences of such a policy would be that governments come to act like overzealous parents, potentially infantilizing autonomous citizens who also and arguably primarily bear responsibility for their own actions. This is aside from the point that governments making substantive judgments about the moral status of the autonomous behavior of citizens, even if positive, will inevitably be controversial. Second, even if one were to accept a wide‐ranging moralization of good persons due to health choices by governments, there is the risk that this will indirectly target and stigmatize those who make other health choices. As such, while praising people for their health behavior in moralized health communications may not always be ethically unacceptable in the way that negative framing is, it is still not desirable for governments to make normative judgments about the kinds of persons who would make certain health choices through moralized health policies.

### Behavior

The second potential target of health moralization is behavior (or actions). A general formulation of health moralizing that targets behavior is as follows: Health‐related behavior(s) *B*(s) is/are morally good or bad.

Focusing on potential behaviors appears to create some distance between normative judgments about health and moral judgments of persons. After all, behaviors can at least theoretically be separated from persons. When it is claimed that getting vaccinated for the sake of others is good, this does not necessarily imply that a person who does not get vaccinated for the sake of others is (therefore) bad. However, in practice, behaviors and the individuals who perform them are very closely related. The connection between health choices and the people who make them will be highly acute, especially for health issues that are controversial and subject to great public scrutiny and debate. The moral conviction that a particular health choice is right or wrong may lead people to view that health choice as indicative of one's moral character,^71^, ^72^ especially when health becomes linked to identity.^73^ In fact, there is evidence that social identity is significantly associated with health‐related behavior.^74^ As such, moralizing health behaviors will, in practice, often amount to moralizing persons, raising all of the previously discussed ethical issues and risks.

Given the intimate ties between health behavior and social identity, moralizing health behavior poses the acute risk that social groups—that is, groups of people who tend on average (or are perceived) to make certain health choices—are singled out, stigmatized, and discriminated against, as was the case for unvaccinated people during the COVID‐19 pandemic.^9^, ^75^ If governments ought not to negatively target individual persons for their health choices, it stands to reason that it should also refrain from doing so for social groups.

What about pragmatic considerations? After all, there is evidence that moral messaging as a framing technique may be more effective than nonmoral messaging in changing people's public health behavior. For instance, Misiak et al.^76^ found that moral messages were more effective than nonmoral messages during the COVID‐19 pandemic in increasing prosocial intentions, particularly when they appealed to heroism. Zhang^77^ found that individuals exposed to a provaccine message framing vaccination as a moral responsibility to protect others from harm—compared to a provaccine message emphasizing self‐protection—reported significantly more positive attitudes toward the flu vaccine as well as an increased intention to get vaccinated. Effectiveness alone clearly does not ethically justify a public health intervention. But perhaps some of the positive consequences associated in the literature with moralizing health behavior may outweigh the risks and harms?

The evidence, however, is not so clear‐cut. Moralizing behavior is often counterproductive and can lead to reactance as well as highly undesirable outcomes, like polarization, stigmatization, and discrimination,^21^, ^26^ which do not benefit health. Furthermore, there is evidence that the use of moral frames can actually “increase and entrench moral divides rather than bridge them” (p. 433)^78^ by lowering people's willingness to compromise, and that nonmoral frames are ultimately more persuasive than moral ones. Moralization can fuel moral outrage and spread via moral contagion,^79^ motivating individuals to harass other people (e.g., in online environments) for punishment and normative reinforcement.^80^ From the perspective of the government, the goal of public health policy should be to protect and promote the health of populations. If moral frames risk causing moral divides for health interventions, as was the case during the COVID‐19 pandemic,^8^, ^9^ then governments ought to avoid using such frames from a pragmatic, if not a moral,^81^ point of view. Health should also not be moralized for political purposes or be weaponized against other people, which is a risk associated more generally with moralizing the behavior of others.^82^

It should furthermore be noted that the evidentiary basis for moralized messaging may not always be sufficiently robust to ethically justify such messaging.^f^ The previously mentioned finding that individuals exposed to a moralized provaccine message for the flu vaccine increased vaccination intentions^77^ should be critically examined in light of the evidence that influenza vaccines (and others, such as COVID‐19 vaccines) are only modestly effective and not very good at preventing the spread of infection to others (and thus preventing harm to others).^83^, ^84^ To morally frame getting vaccinated against the flu (or COVID‐19) as thereby positively protecting others or as thereby ensuring no harm comes to others risks significantly overstating the case and reducing the moral complexity of the matter, which may be incongruent with a duty to fully inform people about the effects of an intervention and may not be ethically justified.^51^

When it comes to positively moralizing health behavior, the same two caveats apply as in the previous case of positively moralizing persons. Compliance with health measures or recommendations does not appear to warrant special praise; nor is it generally the task of governments to praise the good behavior of citizens.^g^ At the same time, there may be room for providing at least some moral context for certain health policies. If a vaccine can prevent or significantly reduce chances of transmission, then using a moral frame that communicates the importance of that fact with the public (i.e., that one might get vaccinated to protect other people) may not be ethically unacceptable. Whether it is ethically desirable or ethically justified, all things considered, is a different matter. Especially when health choices are highly salient, as in the case of vaccination during a pandemic, and binary (i.e., one either gets vaccinated or not), positively moralizing behavior may turn out in practice to be akin to negatively moralizing behavior. That is, the association between a health choice and the moral goodness of that choice may quickly turn into its opposite: the moral badness of the other choice, and of the people who make it.

### Society

The final potential target of health moralization is society (or collective health outcomes). A general formulation of health moralizing that targets society is as follows: Collective health outcome(s) *O*(s) is/are morally good or bad.

Rather than moralizing health by targeting individual persons or behavior, governments might moralize a particular collective health outcome that would benefit everyone in society.^h^ One example is collective protection against infectious diseases. Governments might want to emphasize that achieving robust group‐level protection against an infectious disease stands to protect the health of everyone, including people who are particularly vulnerable to the disease in question. The collective health goal would be to try to increase collective immunity, which might be achieved in various ways, including vaccination.^44^ This line of reasoning seems to be congruent with one of the basic tasks of government, which includes protecting public health and the conditions for societal life given that “the spread of infectious diseases can have severe effects on communal life and protection against such infections is necessary for a flourishing society” (p. 7164).^86^ Attributing this responsibility for public health to states still fits, arguably, within a liberal political conception of government that emphasizes only a modest (rather than a more robust) role for the state.^86^, ^87^

Of course, the argument that governments have a moral responsibility to protect public health and other important societal goods—for instance, as a matter of justice—is not the same as the argument that governments should moralize those public health outcomes or societal goods in communications with the public or through particular health interventions and policies. At the same time, there may be some legitimate space for governments to morally frame measures that would advance or protect collective health goals. From an ethical perspective, moralizing good outcomes for all does not appear to single out any individuals or groups, and could perhaps avoid some of the risks and ethical issues associated with moralizing persons and behavior. Should governments moralize societal health goals?

One important consideration is that collective health goals can only be achieved through individual contributions by citizens. In this sense, one cannot entirely divorce a collective health goal from the individual persons (or behaviors) whose contributions are necessary to achieve that goal. Furthermore, societies are often heterogeneous in terms of not only the efforts that individuals are willing to exert for collective goals, but also in terms of the extent to which the value of the proposed outcome is recognized; people with the highest stakes tend to make the largest contributions.^88^ Public health goals are often collective action problems. If all members of society were to already recognize the moral value of the collective health goal in question and to take the required steps to achieve it, then health interventions—including moral framing—would be needless. It is precisely because there is heterogeneity in the public's acceptance of a health good as a good, in people's willingness and ability to expend efforts to reach a certain health goal, and so on, that government efforts are arguably required. And when it comes to a collective health goal with limited behavioral options to help realize it—for instance, in the case of group protection against infectious diseases through vaccination—the risk is all too real that moralizing that societal good will once again revert, in practice, to targeting the behaviors and the persons that do not contribute to that good. As such, negatively moralized framing of societal health goals is also undesirable.

What about positively moralized health that targets society? Governments need not hide the task that they rightfully have of protecting public health.^89^, ^90^ Trying to achieve a society where good health can be enjoyed by all may align with moral messaging that alerts people to the moral value associated with this aim, especially when it comes to health outcomes that can only or that can best be achieved through collective action—for example, maintaining group‐level immunity against certain infectious diseases, reducing air pollution, and so on. Nevertheless, the risk remains that moralizing good contributions to societal health goals once again turns into a singling out of bad behaviors and persons. As such, the following four conditions should hold for the moralizing of societal health outcomes to be morally acceptable:

1. Government moralizing should not target individual citizens or social groups.
2. Government moralizing should not be excessive—it should not, for instance, overexpose people to moral frames or overstate the moral case.
3. Collective health outcomes moralized by governments should not be unequally achievable—that is, all citizens subjected to moral framing should have the same means, at least in principle, of realizing the health outcome in question.
4. Governments should only moralize health outcomes on the basis of robust evidence that it will bring about the desired outcome (e.g., a healthier society) without being counterproductive or harmful.

The first condition follows from the previous discussions about targeting persons and behavior, and highlights the imperative to avoid stigmatizing and discriminatory effects against individuals and social groups.

The second condition stresses the importance of avoiding excessive moralizing either in terms of the frequency and insistency of moral messaging, which risks creating and exacerbating rather than bridging moral divides.^78^ It also emphasizes that governments should not overstate the moral case for a measure or intervention, for instance by exaggerating the effectiveness of an intervention or by stressing moral reasons or duties that people do not, in fact, have. Excessive moralizing is problematic not least because it undermines the function and downplays the force of legitimate moral criticism.^52^

The third condition is meant to preclude governments positively moralizing societal health outcomes that are achievable only for some citizens, which raises issues of justice. If only a certain group of citizens can, for example, afford to eat healthy fresh food, then population‐wide moralizing about eating healthy food misses the mark. Such a policy, in fact, adds insult to injury for those who would like to eat healthier but do not have the resources to do so—especially if this is due to structural injustices. Similarly, in the case of infectious disease measures, if only a specific group of citizens can practically afford the time and costs of getting vaccinated, then casting the goal of everyone getting vaccinated in moral terms that makes equal demands on all citizens is unfair. Governments should not assume that all citizens are in a similar position to take public health measures upon themselves; when complying with the demands of moralized measures are significantly higher for some individuals or social groups than for others, this is arguably unjust.^91^ In such situations, which are probably much more prevalent than most governments and public health officials care to admit, moralized slogans like “we're all in this together” ring hollow. A single moralized health goal is morally undesirable when it addresses individuals and groups with significantly different capabilities of responding to and ultimately realizing that goal, whether it be negatively or positively framed. Different moral frames might be used for different groups to partly circumvent this issue. However, as I have previously argued, this raises the ethical issue of discrimination between individuals and groups. Of course, ideally, before considering moralizing as a strategy, governments should ensure that all citizens have the practical means of contributing to collective health outcomes.

The fourth and final condition stipulates that governments should consider moral framing only when there is strong evidence for its effectiveness. This condition is meant to ensure that moralizing will be efficacious and will achieve the desired health outcome (e.g., better collective health)—without, for instance, being counterproductive or causing harmful effects. For it is not straightforwardly the case that moral framing and moral motives always lead to better outcomes. Prosocial motives have, for example, been found to underlie scientific censorship by scientists,^92^ which is arguably an undesirable outcome for society in the long run. Some of these effects may be counterintuitive, hence the need for evidence‐based policy. When an issue is moralized, there are other potentially harmful effects to consider, like the propensity for individuals to moralistically punish others,^93^ which governments ought not to encourage. Substantial evidence of effectiveness is a basic requirement when it comes to moralizing collective health outcomes, to ensure that health and other important societal goals—like avoiding social strife—are actually advanced rather than frustrated.

## CONCLUSION

This paper addressed the question of whether governments should moralize health. It did so by categorizing different aspects that affect its moral acceptability. Two distinctions were made: first, between negative and positive framing, and second, between different potential targets toward which moralizing may be directed (i.e., persons, behavior, or society). The overall conclusion of the arguments presented in this paper is that health moralizing that targets individual persons and behavior, especially in negative ways, is morally unacceptable. Positive moralizing about collective health outcomes may be more acceptable, but conditions for its moral acceptability remain. Governments should not target individuals or social groups, in order to avoid the risk of stigmatization. Moralizing must not be excessive and should be based on robust evidence that it will bring about the desired outcome (i.e., a healthier society) without being harmful or counterproductive. Finally, if collective health outcomes are to be moralized, then all citizens should be practically able to meet the demands of those outcomes as a matter of justice. While much theoretical and empirical work remains to be done on the nature and ethics of moralizing health—both in general and in relation to the specific role of governments—I hope to have provided a useful starting point and argumentative structure for future discussions.

## CONFLICT OF INTEREST STATEMENT

The author declares no conflicts of interest.

## PEER REVIEW

The peer review history for this article is available at https://publons.com/publon/10.1111/nyas.70030.

## Data Availability

Data sharing is not applicable to this article as no new data were created or analyzed in this study.

## Footnotes

a Throughout this paper, *governments* is used as a broad term that includes not only official government policies, regulations, and communications, but also any public official acting in a government capacity (e.g., a politician, a government‐employed scientist, etc.).

b It should be noted that the harms at stake, including their severity and moral significance, cannot always be clearly determined beforehand and that their (moral) weight also be subject to reasonable disagreement, which is an important factor to consider in discussions about, for example, infectious disease ethics and policy.^21^, ^51^

c Nor does it entail that all behavior that potentially harms others is intrinsically morally wrong.

d While recognizing that the explicitness (or subtlety) of moral framing and associated implications may be ethically relevant, limitations of space preclude further addressing this matter here. Future research should address the matter.

e Granted, consequentialist theories also refer to societal or global outcomes, like the maximization of the greatest good for all sentient beings, but it is still most directly concerned with how an individual ought to *act* (i.e., to achieve that outcome).^63^

^f^
Thanks to an anonymous reviewer for raising this point.

g There may be some legitimate room for governments to commend exemplary or even heroic behavior, perhaps especially during times of crisis. I do not take a strong stand on this matter here. As I argued earlier in the paper (in the section on *Persons*), most health behaviors in any case do not easily lend themselves to heroic acts—nor, accordingly, to heroic praise.

h It should be noted that moral framing of health measures and policy, like any other policy, is a political choice subject to changes in political administrations. Kreitzer, Kane, and Mooney, for instance, have argued that no public policy is inherently moral, and that “policies may be 'moralized” or 'demoralized' over time, not due to any intrinsic characteristic, but because the prominent policy frames in their debate have changed' (p. 3).^85^ The frames in question are precisely what is at stake in the present discussion.
